# Phosphorylation of Pyruvate Dehydrogenase as a Marker of Neuronal Inactivity in the Enteric Nervous System

**DOI:** 10.1111/nmo.70135

**Published:** 2025-08-16

**Authors:** Eve Wemelle, Jonathon L. McClain, Brian D. Gulbransen

**Affiliations:** ^1^ Department of Physiology Michigan State University East Lansing Michigan USA

**Keywords:** enteric neurons, inactivation, markers

## Abstract

**Background:**

The enteric nervous system (ENS) regulates essential gut functions through interactions between neurons and glial cells. While tools for studying neuronal activation are well‐established, methods for tracking neuronal inactivation remain underdeveloped. Phosphorylated pyruvate dehydrogenase (pPDH) has emerged as a marker of neuronal inactivity in the brain. This study investigates whether pPDH can similarly serve as a reliable marker of neuronal inactivity in the ENS.

**Methods:**

Whole‐mount preparations of the colonic myenteric plexus from mice were treated with veratridine (neuronal activator) or tetrodotoxin (neuronal inhibitor). Immunohistochemistry was used to label neurons with HuC/D and quantify pPDH (inactivity marker) and pERK (activation marker) labelling. Fluorescent signals were analyzed to assess changes in marker expression under different conditions.

**Results:**

TTX treatment increased pPDH labelling, as evidenced by higher fluorescence intensity and a greater percentage of pPDH‐positive neurons. In contrast, veratridine reduced pPDH levels, indicating its sensitivity to neuronal activity changes. Together, pPDH and pERK provide reliable measures of neuronal inactivation and activation, respectively, in the ENS.

**Conclusions:**

This study establishes pPDH as a robust marker for neuronal inactivation in the ENS, complementing existing activation markers like pERK. These findings provide a novel framework for exploring neuron–glia communication and neuronal dynamics in the gut.

## Graphical abstract

Phosphorylated pyruvate dehydrogenase (pPDH) serves as a novel histological marker of neuronal inactivation in the enteric nervous system. Pharmacological modulation of neuronal activity using TTX and veratridine revealed that pPDH levels are inversely correlated with neuronal firing. In contrast, pERK expression followed the opposite trend, validating the activity dependence of pPDH.

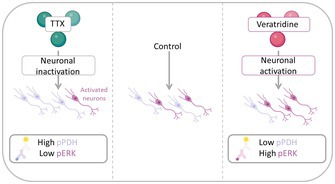

## 1 Introduction

The enteric nervous system (ENS) orchestrates essential gut functions, such as motility, secretion, and local reflexes through intrinsic circuitry composed of neurons and glial cells [1]. Understanding the dynamics of enteric neuron activation and inactivation is therefore fundamental to understanding gut functions in health and disease. As a result, numerous techniques have been developed to study neuronal activity in the gut. These include measuring the expression of molecular markers, such as the immediate early gene *c‐Fos* that are increased in active cells, using genetically encoded or organic sensors for voltage and mediators of cell activity, such as calcium to record activity, and electrophysiological recordings that provide detailed single‐cell level insights into ion channel dynamics and action potential firing [2, 3]. Therefore, investigators have a good selection of approaches that provide reliable readouts of neuronal activation. In contrast, comparable methods to label and study neuronal inactivation in the gut remain underexplored. Given that patterns of neuron inactivity are fundamental to ENS‐controlled functions such as the colonic motor complex, simple and reliable methods to study neuron inactivity could be a useful addition to the ENS toolbox.

Phosphorylated pyruvate dehydrogenase (pPDH) recently emerged as a robust inverse activity marker in a comprehensive phosphoproteomic screen of markers of cell inactivity in the brain, and was subsequently validated by several approaches including optogenetics and chemogenetics [4]. pPDH inversely correlates with neuronal firing and provides a stable molecular signature of reduced activity. Inspired by these findings, we sought to investigate whether pPDH could similarly serve as a reliable marker of neuronal inactivation in the ENS. To this end, we used veratridine, a known activator of voltage‐gated sodium channels [5], to induce neuronal activation and tetrodotoxin (TTX) [6], a sodium channel blocker, to reduce activity among myenteric neurons in the colon. Results from subsequent labelling with pERK to mark activated neurons and pPDH to label inactivated neurons show that these markers offer reliable inverse measures of neuronal dynamics in the ENS. Based on these observations, we propose that pPDH offers a useful tool for studying neuronal inactivity in the ENS.

## 2 Material and Methods

### 2.1 Animals

All animal experiments were performed in compliance with the guidelines outlined in the National Institutes of Health (NIH) Guide for the Care and Use of Laboratory Animals and received approval (PROTO202400108) from the Institutional Animal Care and Use Committee (IACUC) at Michigan State University (MSU). Unless otherwise specified, two male and three female mice aged 8–11 weeks and of the C57BL/6 genetic background were utilized. The mice were housed under a 12‐h light/dark cycle in a temperature‐controlled environment and had unrestricted access to water and standard laboratory chow (Diet Number 2919; Envigo, Indianapolis, IN).

### 2.2 Immunohistochemistry

Whole‐mount preparations of the distal colonic myenteric plexus were prepared by microdissecting the mucosa, submucosa, and circular muscle layers. The resulting longitudinal muscle‐myenteric plexus (LMMP) whole‐mounts were incubated with TTX (1 μM, Calbiochem, Merck KGaA, Darmstadt, Germany), Veratridine (10 μM, Tocris, BioTechne, Minneapolis, MN), or DMEM (CT) for 30 min and then fixed in 4% paraformaldehyde (PFA) for 1 h at room temperature before being processed for whole‐mount immunohistochemistry as described in previous studies [7]. Samples were washed with 0.1% Triton X‐100 in PBS (3 × 10 min each) and then incubated for 45 min in a blocking solution containing 4% normal donkey serum, 0.4% Triton X‐100, and 1% bovine serum albumin. Samples were then incubated with primary antibodies overnight at 4°C followed by secondary antibody incubation for 2 h at room temperature. Finally, samples were mounted on slides using DAPI Fluoromount (Fisher Scientific, Carlsbad, CA) for nuclear staining and long‐term preservation. Fluorescent labelling was visualized using a Nikon Ni‐E Microscope equipped with a Nikon AX Confocal system. Imaging was performed through the ApoLWD 25 × 1.10 WDIC N2 water immersion objective (numerical aperture, NA = 1.1), providing high resolution and optimal light collection for confocal imaging (resolution 1024 × 1024 with line averaging of 2). The microscope settings were adjusted to optimize the signal‐to‐noise ratio, ensuring precise detection of fluorescence signals for all markers of interest. For each mouse, images were acquired with two ganglia per frame, and two frames were analyzed per animal.

**Table:** 

Antibody	Source	Catalog numbers	Dilution	RRIDs
pERK	Cell Signaling	4370S	1/200	AB_2315112
pPDH	Cell Signaling	37115	1/1000	AB_2923272
HuC/D	Thermo Fisher	A21272	1/200	AB_2535822
Donkey anti‐Rabbit Alexa Fluor 647	Jackson	711‐605‐152	1/400	AB_2492288
Goat anti‐Mice IgG2b Alexa Fluor 594	Jackson	115‐585‐207	1/400	AB_2338887

### 2.3 Analysis and Statistics

Analyses were performed for each image in a blinded manner. The fluorescence intensity per cell was quantified, and positively labeled cells (raw fluorescent ≥ median of all condition) were manually counted using FIJI (National Institutes of Health) with the cell counter analysis plugin. No sex differences were observed, so data from males and females were pooled together for analysis. Statistical analyses were conducted using one‐way ANOVA or a Kruskal–Wallis test (for the % of positive cells) in GraphPad Prism 10. Data are expressed as mean ± standard error of the mean (SEM). Outliers were identified and excluded using the ROUT method (*Q* = 1%) in GraphPad.

## 3 Results

Neuronal firing is an energy‐intensive process that is primarily supported by oxidative metabolism. Activation of the tricarboxylic acid (TCA) cycle is essential for neuronal activity in the brain, as it provides the ATP required to meet the high energy demands of active neurons [8]. Pyruvate dehydrogenase (PDH) plays a critical role in this process by catalyzing the conversion of pyruvate into acetyl‐CoA, a substrate for the TCA cycle. Phosphorylation of PDH inhibits this enzymatic activity. Conversely, dephosphorylation of PDH reactivates its enzymatic function, ensuring sufficient ATP generation to support neuronal firing and associated metabolic demands. To determine whether PDH phosphorylation could serve as a reliable indicator of neuronal activity in the gut, as it does in the brain, we took advantage of the spontaneous activity of gut neurons [9]. We used two different drugs to modulate neuronal activity: TTX, to block neuronal firing, and veratridine, to activate it. Based on findings from brain studies, we selected a 30‐min incubation period, which corresponds to the timeframe required to achieve maximal PDH phosphorylation in neuronal tissues [4].

**FIGURE 1:**
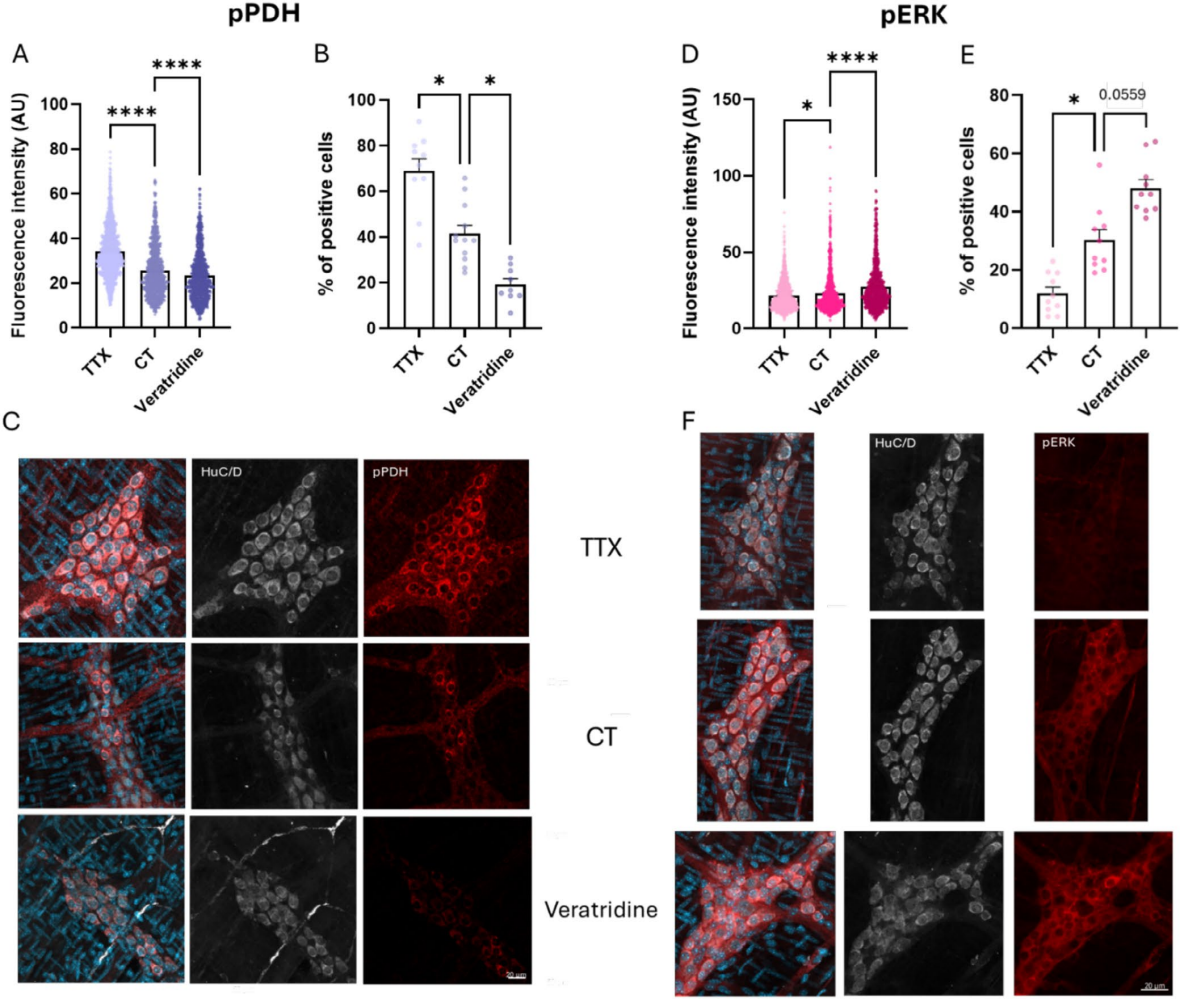
Quantification of raw fluorescence (1 dot/cell) and percentage of positive cells (1 dot/frame) for pPDH (A, B) and pERK (D, E) labelling, in neurons. Representative images (maximal intensity of a *Z*‐stack: 8 frames of 1 μm) of pPDH (C), pERK (F) with HuC/D (neuronal marker) labelling with TTX, CT or Veratridine treatment (scale = 20 μm). Significant differences were determined by ANOVA one‐way and Kruskal–Wallis analysis (*p < 0.05, ****p < 0.0001).

We identified the cells as neurons using HuC/D labelling, a well‐established neuronal marker that specifically labels neuronal nuclei and cytoplasm. This approach ensured that the observed changes in pPDH levels were indeed occurring in gut neurons. Results show that PDH phosphorylation levels in gut neurons are modulated by neuronal activity. Specifically, treatment with TTX, which suppresses neuronal firing, significantly increased pPDH levels, as evidenced by both a higher raw fluorescence intensity per cell (34.23 ± 0.30 AU, Figure 1A,C) and an increase in the percentage of pPDH‐positive cells (69% ± 5% Figure 1B). In contrast, veratridine, which enhances neuronal firing, caused a substantial decrease in both the fluorescence intensity (23.27 ± 0.32 AU, Figure 1A,C) and the percentage of pPDH‐positive cells (19% ± 3%, Figure 1B).

To confirm these observations, we evaluated pERK, a well‐established marker of cell activation. pERK levels were inversely correlated with pPDH levels. TTX treatment resulted in a significant reduction in raw fluorescence intensity (21.57 ± 0.31 AU, Figure 1D,F) and the percentage of pERK‐positive cells (12% ± 2%, Figure 1E). Conversely, veratridine induced a robust increase in both the percentage of pERK‐positive cells (48% ± 3%, Figure 1E) and fluorescence intensity (27.33 ± 0.45 AU, Figure 1D,F). These results suggest that pERK can serve as a reliable marker of neuronal activation in the gut, and its inverse relationship with pPDH provides additional support for the validity of PDH phosphorylation as a dynamic and activity‐dependent marker of neuronal inactivation.

Together, these findings underline the reciprocal dynamics of pPDH and pERK as markers of neuronal inactivation and activation, respectively, providing a complementary framework for studying neuronal states in the gut.

## 4 Discussion

Neuronal dynamics are characterized by both increases and decreases in activity, yet much of the past work on the ENS has predominantly focused on tracking neuronal activation because monitoring and quantifying reduced neuronal activity with traditional markers has remained a significant challenge. This imbalance in the tools available to researchers highlights the critical need for novel methodologies to profile, track, and study not just the active states of neurons, but also their periods of inactivation. Neuronal inactivity is a key aspect of circuit dynamics and influences critical functions, such as gastrointestinal motility, secretion, or local reflexes. Furthermore, changes in the balance between activation and inactivation states may underlie disease conditions like irritable bowel syndrome (IBS) [10], where dysregulated neuronal signaling contributes to altered gut function. Measuring inactivity, however, is inherently challenging with traditional techniques, such as Ca^2+^ imaging, which are designed to detect the dynamic calcium influxes associated with neuronal activation but provide limited insight into quiescent or inactive states. Developing reliable markers of neuronal inactivity, such as pPDH, opens new perspectives for studying both physiological processes and pathophysiological conditions in the enteric nervous system.

A limitation of this study is the lack of direct functional confirmation of neuronal inactivity through techniques like electrophysiological recordings, which are technically demanding and beyond the scope of this report. Nonetheless, the use of pharmacological tools with well‐established effects (veratridine and TTX) provides strong support for the relevance of pPDH as a marker of neuronal inactivity in the ENS.

Based on a new approach published in the brain [4], we showed that pPDH phosphorylation reliably tracks neuronal inactivation in the gut, providing a valuable tool to study the molecular mechanisms underlying neuron–glia communication and their roles in health and disease. Specifically, these biomarkers will be useful to identify new molecules that promote neuronal inactivity, which could be explored as therapeutic strategies for diseases involving neuronal hyperactivation, such as diarrhea or chronic intestinal pseudo‐obstruction (CIPO).

## Author Contributions

Study concept and design: E.W. and B.D.G. Acquisition of data: E.W. and J.L.M. Analysis and interpretation of data: E.W. Drafting of the manuscript: E.W. Critical revision of the manuscript: E.W., J.L.M., and B.D.G. Statistical analysis: E.W. Obtained funding: B.D.G.

## Conflicts of Interest

The authors declare no conflicts of interest.

## Data Availability

The data that support the findings of this study are available from the corresponding author upon reasonable request.
